# (*E*)-1-(4,4′′-Difluoro-5′-meth­oxy-1,1′:3′,1′′-terphenyl-4′-yl)-3-(4-meth­oxy­phen­yl)prop-2-en-1-one

**DOI:** 10.1107/S1600536811047696

**Published:** 2011-11-16

**Authors:** Richard Betz, Thomas Gerber, Eric Hosten, S. Samshuddin, Badiadka Narayana, Hemmige S. Yathirajan

**Affiliations:** a Nelson Mandela Metropolitan University, Summerstrand Campus, Department of Chemistry, University Way, Summerstrand, PO Box 77000, Port Elizabeth 6031, South Africa; b Mangalore University, Department of Studies in Chemistry, Mangalagangotri 574 199, India; c University of Mysore, Department of Studies in Chemistry, Manasagangotri, Mysore 570 006, India

## Abstract

The title compound, C_29_H_22_F_2_O_3_, is a *meta*-terphenyl derivative featuring a Michael-system-derived substituent with an *E*-configured C=C function. In the crystal, C—H⋯O and C—H⋯F contacts connect the mol­ecules into planes parallel to (101). The shortest centroid–centroid distance between two aromatic systems is 3.7169 (7) Å and is apparent between the terminal benzene ring of the Michael-system-derived substituent and its symmetry-generated equivalent.

## Related literature

For the pharmacological importance of terphenyls, see: Liu (2006 ▶) and of chalcones, see: Dhar (1981 ▶); Dimmock *et al.* (1999 ▶); Satyanarayana *et al.* (2004 ▶). For our work on the synthesis of different chalcone derivatives, see: Samshuddin *et al.* (2011 ▶); Fun *et al.* (2010 ▶); Jasinski *et al.* (2010 ▶); Baktır *et al.* (2011 ▶). For graph-set analysis of hydrogen bonds, see: Etter *et al.* (1990 ▶); Bernstein *et al.* (1995 ▶).
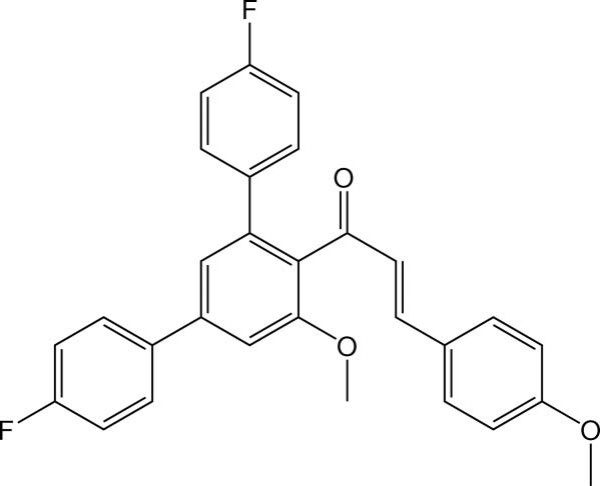


## Experimental

### 

#### Crystal data

C_29_H_22_F_2_O_3_
*M* *_r_* = 456.47
Monoclinic, 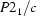

*a* = 9.6059 (2) Å
*b* = 19.2236 (5) Å
*c* = 13.3772 (3) Å
β = 112.905 (1)°
*V* = 2275.46 (9) Å^3^
*Z* = 4
Mo *K*α radiation
μ = 0.10 mm^−1^
*T* = 200 K
0.54 × 0.51 × 0.51 mm

#### Data collection

Bruker APEXII CCD diffractometer
20209 measured reflections
5655 independent reflections
4754 reflections with *I* > 2σ(*I*)
*R*_int_ = 0.032

#### Refinement

*R*[*F* ^2^ > 2σ(*F* ^2^)] = 0.045
*wR*(*F* ^2^) = 0.125
*S* = 1.03
5655 reflections
309 parameters
H-atom parameters constrained
Δρ_max_ = 0.37 e Å^−3^
Δρ_min_ = −0.28 e Å^−3^

### 

Data collection: *APEX2* (Bruker, 2010 ▶); cell refinement: *SAINT* (Bruker, 2010 ▶); data reduction: *SAINT*; program(s) used to solve structure: *SHELXS97* (Sheldrick, 2008 ▶); program(s) used to refine structure: *SHELXL97* (Sheldrick, 2008 ▶); molecular graphics: *ORTEP-3* (Farrugia, 1997 ▶) and *Mercury* (Macrae *et al.*, 2008 ▶); software used to prepare material for publication: *SHELXL97* and *PLATON* (Spek, 2009 ▶).

## Supplementary Material

Crystal structure: contains datablock(s) I, global. DOI: 10.1107/S1600536811047696/qk2026sup1.cif

Supplementary material file. DOI: 10.1107/S1600536811047696/qk2026Isup2.cdx

Structure factors: contains datablock(s) I. DOI: 10.1107/S1600536811047696/qk2026Isup3.hkl

Supplementary material file. DOI: 10.1107/S1600536811047696/qk2026Isup4.cml

Additional supplementary materials:  crystallographic information; 3D view; checkCIF report

## Figures and Tables

**Table 1:** Hydrogen-bond geometry (Å, °)

*D*—H⋯*A*	*D*—H	H⋯*A*	*D*⋯*A*	*D*—H⋯*A*
C3—H3⋯F1^i^	0.95	2.51	3.4159 (15)	159
C5—H5*B*⋯O1^ii^	0.98	2.54	3.3534 (18)	141
C22—H22⋯O1^iii^	0.95	2.51	3.4208 (18)	161

Symmetry codes: (i) 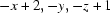; (ii) 
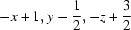
; (iii) 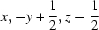.

**Fig. 1.**
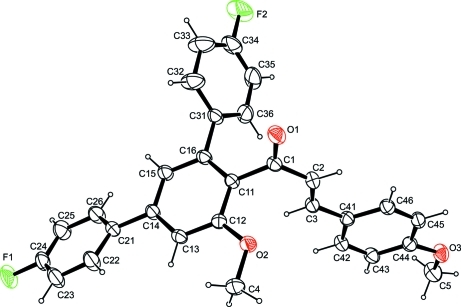
The molecular structure of the title compound, with atom labels and anisotropic displacement ellipsoids (drawn at 50% probability level).

**Fig. 2.**
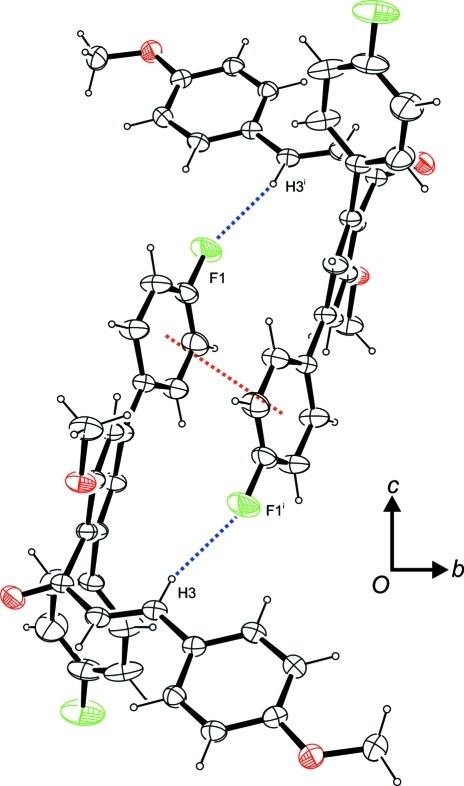
Intermolecular contacts, viewed along [-1 0 0]. For clarity, only C–H···F contacts (blue dashed lines) as well as a π···π interaction (red dashed line, Cg2—Cg2i = 3.9381 (9) Å) are shown. Symmetry operator: i -x + 2, -y, -z + 1.

## supplementary crystallographic information

### Comment

Chalcones constitute an important family of substances belonging to flavonoids, a large group of natural and synthetic products with interesting physicochemical properties, biological activity and structural characteristics. They have been reported to possess many interesting pharmacological activities (Dhar, 1981) including anti-inflammatory, antimicrobial, antifungal, antioxidant, cytotoxic, antitumor and anticancer activities (Dimmock *et al.*, 1999; Satyanarayana *et al.*, 2004). In recent years, it has been reported that some terphenyls exhibit considerable biological activities (*e.g.* being potent anticoagulants, immunosuppressants, antithrombotics, neuroprotectives, specific 5-lipoxygenase inhibitors) and showing cytotoxic activities (Liu, 2006). In view of the pharmacological importance of terphenyls and chalcones, and in continuation of our work on synthesis of various derivatives of 4,4'-difluoro chalcone (Samshuddin *et al.*, 2011, Fun *et al.*, 2010, Jasinski *et al.*, 2010, Baktır *et al.*, 2011), the molecular and crystal structure of the title compound is reported.

The C=C function along the Michael system is (*E*)-configured. The least-squares planes defined by the respective carbon atoms of the individual *para*-fluorophenyl moieties enclose angles of 25.42 (5) ° and 64.01 (5) ° with the plane defined by the carbon atoms of the terphenyl's central phenyl ring.

In the crystal, C–H···O as well as C–H···F contacts can be observed whose range falls by more than 0.1 Å below the sum of van-der-Waals radii of the respective atoms. The C–H···O contacts stem from one of the hydrogen atoms of a *para*-fluoro phenyl moiety and one of the methoxy groups and invariably apply the ketonic oxygen atom as acceptor. The C–H···F contacts are exclusively supported by the vinylic hydrogen atom on the Michael system. In terms of graph-set analysis (Etter *et al.*, 1990; Bernstein *et al.*, 1995), the descriptor for the C–H···F contacts is *R*^2^_2_(26) on the unitary level while the C–H···O contacts necessitate a *C*^1^_1_(9)*C*^1^_1_(10) descriptor on the same level. In total, the molecules are connected to planes parallel (1 0 1). The shortest intercentroid distance between two ring centroids was found at 3.7169 (7) Å and is apparent between the methoxyphenyl moiety connected to the Michael system and its symmetry-generated equivalents. Details about metrical parameters of the C–H···O and C–H···F contacts as well as information about their symmetry can be found in Table 1 (Fig. 2).

### Experimental

To a mixture of 1-(4,4''-difluoro-5'-methoxy-1,1':3',1''-terphenyl-4'-yl) ethanone (3.38 g, 0.01 mol) and anisaldehyde (1.36 g, 0.01 mol) in 30 ml e thanol, 10 ml of 10% sodium hydroxide solution was added and stirred at 5–10 °C for 3 h. The precipitate formed was collected by filtration and purified by recrystallization from ethanol (yield: 79%). Single crystals suitable for the X-ray diffraction study were grown from DMF by slow evaporation at room temperature.

### Refinement

Carbon-bound H atoms were placed in calculated positions (C—H 0.95 Å and C—H 0.98 Å for the methyl groups, *see below*) and were included in the refinement in the riding model approximation, with *U*(H) set to 1.2*U*_eq_(C). The H atoms of the methyl groups were allowed to rotate with a fixed angle around the C—C bond to best fit the experimental electron density (HFIX 137 in the *SHELX* program suite (Sheldrick, 2008)), with *U*(H) set to 1.5*U*_eq_(C).

### Crystal data

**Table:**

C_29_H_22_F_2_O_3_	*F*(000) = 952
*M**_r_* = 456.47	*D*_x_ = 1.332 Mg m^−^^3^
Monoclinic, *P*2_1_/*c*	Melting point: 453 K
Hall symbol: -P 2ybc	Mo *K*α radiation, λ = 0.71073 Å
*a* = 9.6059 (2) Å	Cell parameters from 9984 reflections
*b* = 19.2236 (5) Å	θ = 2.3–28.3°
*c* = 13.3772 (3) Å	µ = 0.10 mm^−^^1^
β = 112.905 (1)°	*T* = 200 K
*V* = 2275.46 (9) Å^3^	Block, colourless
*Z* = 4	0.54 × 0.51 × 0.51 mm

### Data collection

**Table:**

Bruker APEXII CCD diffractometer	4754 reflections with *I* > 2σ(*I*)
Radiation source: fine-focus sealed tube	*R*_int_ = 0.032
graphite	θ_max_ = 28.3°, θ_min_ = 2.0°
φ and ω scans	*h* = −12→12
20209 measured reflections	*k* = −25→16
5655 independent reflections	*l* = −17→17

### Refinement

**Table:**

Refinement on *F*^2^	Primary atom site location: structure-invariant direct methods
Least-squares matrix: full	Secondary atom site location: difference Fourier map
*R*[*F*^2^ > 2σ(*F*^2^)] = 0.045	Hydrogen site location: inferred from neighbouring sites
*wR*(*F*^2^) = 0.125	H-atom parameters constrained
*S* = 1.03	*w* = 1/[σ^2^(*F*_o_^2^) + (0.0584*P*)^2^ + 0.9094*P*] where *P* = (*F*_o_^2^ + 2*F*_c_^2^)/3
5655 reflections	(Δ/σ)_max_ < 0.001
309 parameters	Δρ_max_ = 0.37 e Å^−^^3^
0 restraints	Δρ_min_ = −0.28 e Å^−^^3^

### Fractional atomic coordinates and isotropic or equivalent isotropic displacement parameters (Å^2^)

**Table:**

	*x*	*y*	*z*	*U*_iso_*/*U*_eq_	
F1	1.26002 (12)	0.02346 (5)	0.34310 (8)	0.0531 (3)	
F2	1.29595 (14)	0.18370 (8)	1.21317 (8)	0.0771 (4)	
O1	0.72100 (12)	0.25739 (5)	0.81288 (8)	0.0372 (2)	
O2	0.56634 (10)	0.17164 (5)	0.56481 (7)	0.0331 (2)	
O3	0.22589 (11)	−0.09964 (5)	0.91422 (8)	0.0364 (2)	
C1	0.70335 (13)	0.19685 (6)	0.78248 (9)	0.0255 (2)	
C2	0.59846 (14)	0.15198 (7)	0.80742 (10)	0.0295 (3)	
H2	0.5335	0.1731	0.8369	0.035*	
C3	0.58766 (13)	0.08347 (7)	0.79165 (10)	0.0271 (2)	
H3	0.6480	0.0636	0.7572	0.033*	
C4	0.48881 (15)	0.16369 (9)	0.45031 (11)	0.0394 (3)	
H4A	0.4974	0.1154	0.4301	0.059*	
H4B	0.5338	0.1947	0.4129	0.059*	
H4C	0.3820	0.1756	0.4293	0.059*	
C5	0.23701 (19)	−0.17318 (8)	0.90367 (14)	0.0429 (3)	
H5A	0.3431	−0.1874	0.9382	0.064*	
H5B	0.1975	−0.1857	0.8266	0.064*	
H5C	0.1780	−0.1969	0.9390	0.064*	
C11	0.79205 (13)	0.16861 (6)	0.71955 (9)	0.0236 (2)	
C12	0.71812 (13)	0.15845 (6)	0.60728 (9)	0.0245 (2)	
C13	0.79734 (13)	0.13784 (7)	0.54504 (9)	0.0262 (2)	
H13	0.7452	0.1310	0.4692	0.031*	
C14	0.95320 (13)	0.12709 (6)	0.59309 (9)	0.0257 (2)	
C15	1.02729 (13)	0.13882 (7)	0.70402 (10)	0.0283 (3)	
H15	1.1338	0.1330	0.7372	0.034*	
C16	0.94848 (13)	0.15899 (6)	0.76765 (9)	0.0256 (2)	
C21	1.03663 (13)	0.10142 (7)	0.52704 (10)	0.0265 (2)	
C22	0.98189 (16)	0.11293 (9)	0.41562 (11)	0.0384 (3)	
H22	0.8925	0.1396	0.3816	0.046*	
C23	1.05596 (18)	0.08598 (9)	0.35336 (12)	0.0435 (4)	
H23	1.0168	0.0929	0.2771	0.052*	
C24	1.18607 (17)	0.04932 (8)	0.40404 (12)	0.0366 (3)	
C25	1.24621 (17)	0.03782 (8)	0.51371 (12)	0.0404 (3)	
H25	1.3383	0.0130	0.5472	0.048*	
C26	1.16917 (15)	0.06331 (8)	0.57462 (11)	0.0353 (3)	
H26	1.2078	0.0545	0.6505	0.042*	
C31	1.03597 (13)	0.16740 (7)	0.88673 (9)	0.0290 (3)	
C32	1.1509 (2)	0.21657 (9)	0.92460 (12)	0.0494 (4)	
H32	1.1697	0.2463	0.8745	0.059*	
C33	1.2391 (2)	0.22275 (11)	1.03526 (14)	0.0601 (5)	
H33	1.3168	0.2567	1.0615	0.072*	
C34	1.21002 (18)	0.17847 (11)	1.10472 (11)	0.0501 (4)	
C35	1.10140 (19)	0.12816 (12)	1.07091 (12)	0.0538 (5)	
H35	1.0867	0.0973	1.1214	0.065*	
C36	1.01272 (16)	0.12334 (10)	0.96049 (11)	0.0442 (4)	
H36	0.9351	0.0893	0.9354	0.053*	
C41	0.49220 (13)	0.03587 (6)	0.82201 (9)	0.0261 (2)	
C42	0.49079 (14)	−0.03467 (7)	0.79823 (10)	0.0298 (3)	
H42	0.5519	−0.0509	0.7618	0.036*	
C43	0.40287 (15)	−0.08220 (7)	0.82603 (10)	0.0310 (3)	
H43	0.4035	−0.1301	0.8086	0.037*	
C44	0.31392 (14)	−0.05848 (7)	0.87982 (10)	0.0282 (2)	
C45	0.31186 (14)	0.01215 (7)	0.90335 (10)	0.0285 (3)	
H45	0.2493	0.0283	0.9387	0.034*	
C46	0.39985 (13)	0.05836 (7)	0.87571 (10)	0.0272 (2)	
H46	0.3985	0.1062	0.8930	0.033*	

### Atomic displacement parameters (Å^2^)

**Table:**

	*U*^11^	*U*^22^	*U*^33^	*U*^12^	*U*^13^	*U*^23^
F1	0.0621 (6)	0.0616 (6)	0.0549 (6)	0.0049 (5)	0.0437 (5)	−0.0110 (5)
F2	0.0674 (7)	0.1271 (12)	0.0192 (4)	0.0025 (7)	−0.0025 (4)	−0.0068 (5)
O1	0.0469 (6)	0.0328 (5)	0.0397 (5)	−0.0057 (4)	0.0254 (4)	−0.0092 (4)
O2	0.0227 (4)	0.0522 (6)	0.0219 (4)	0.0035 (4)	0.0059 (3)	−0.0026 (4)
O3	0.0441 (5)	0.0325 (5)	0.0414 (5)	−0.0044 (4)	0.0263 (4)	−0.0002 (4)
C1	0.0262 (5)	0.0306 (6)	0.0199 (5)	0.0001 (4)	0.0093 (4)	−0.0011 (4)
C2	0.0301 (6)	0.0349 (7)	0.0283 (6)	0.0000 (5)	0.0168 (5)	−0.0023 (5)
C3	0.0258 (5)	0.0345 (6)	0.0229 (5)	0.0010 (5)	0.0115 (4)	−0.0011 (5)
C4	0.0257 (6)	0.0624 (9)	0.0237 (6)	0.0019 (6)	0.0027 (5)	−0.0031 (6)
C5	0.0580 (9)	0.0317 (7)	0.0477 (8)	−0.0039 (6)	0.0301 (7)	0.0011 (6)
C11	0.0260 (5)	0.0260 (5)	0.0197 (5)	−0.0005 (4)	0.0100 (4)	−0.0003 (4)
C12	0.0222 (5)	0.0294 (6)	0.0211 (5)	−0.0003 (4)	0.0074 (4)	0.0001 (4)
C13	0.0271 (5)	0.0329 (6)	0.0173 (5)	0.0003 (5)	0.0073 (4)	−0.0011 (4)
C14	0.0271 (5)	0.0306 (6)	0.0207 (5)	0.0018 (4)	0.0107 (4)	0.0003 (4)
C15	0.0235 (5)	0.0380 (7)	0.0220 (5)	0.0036 (5)	0.0072 (4)	−0.0008 (5)
C16	0.0271 (5)	0.0302 (6)	0.0183 (5)	0.0010 (4)	0.0077 (4)	0.0001 (4)
C21	0.0279 (5)	0.0313 (6)	0.0221 (5)	−0.0005 (5)	0.0115 (4)	−0.0021 (4)
C22	0.0366 (7)	0.0549 (9)	0.0268 (6)	0.0100 (6)	0.0159 (5)	0.0044 (6)
C23	0.0479 (8)	0.0624 (10)	0.0267 (6)	0.0050 (7)	0.0216 (6)	0.0004 (6)
C24	0.0436 (7)	0.0390 (7)	0.0387 (7)	−0.0023 (6)	0.0286 (6)	−0.0078 (6)
C25	0.0364 (7)	0.0469 (8)	0.0411 (8)	0.0104 (6)	0.0187 (6)	−0.0023 (6)
C26	0.0341 (6)	0.0456 (8)	0.0269 (6)	0.0074 (6)	0.0124 (5)	−0.0008 (5)
C31	0.0257 (5)	0.0408 (7)	0.0194 (5)	0.0054 (5)	0.0074 (4)	−0.0016 (5)
C32	0.0554 (9)	0.0532 (9)	0.0264 (7)	−0.0139 (7)	0.0017 (6)	0.0016 (6)
C33	0.0619 (11)	0.0666 (12)	0.0312 (8)	−0.0165 (9)	−0.0042 (7)	−0.0058 (8)
C34	0.0407 (8)	0.0827 (13)	0.0188 (6)	0.0093 (8)	0.0028 (5)	−0.0045 (7)
C35	0.0420 (8)	0.0962 (14)	0.0243 (7)	0.0009 (8)	0.0143 (6)	0.0119 (8)
C36	0.0336 (7)	0.0730 (11)	0.0255 (6)	−0.0064 (7)	0.0110 (5)	0.0056 (7)
C41	0.0255 (5)	0.0307 (6)	0.0223 (5)	0.0013 (4)	0.0095 (4)	0.0003 (4)
C42	0.0323 (6)	0.0332 (6)	0.0282 (6)	0.0015 (5)	0.0165 (5)	−0.0032 (5)
C43	0.0364 (6)	0.0282 (6)	0.0306 (6)	0.0000 (5)	0.0154 (5)	−0.0034 (5)
C44	0.0281 (6)	0.0323 (6)	0.0243 (6)	−0.0012 (5)	0.0102 (4)	0.0020 (5)
C45	0.0274 (5)	0.0346 (6)	0.0260 (6)	0.0039 (5)	0.0130 (5)	0.0006 (5)
C46	0.0277 (5)	0.0285 (6)	0.0259 (6)	0.0032 (4)	0.0109 (4)	−0.0004 (5)

### Geometric parameters (Å, °)

**Table:**

F1—C24	1.3663 (14)	C21—C22	1.3917 (17)
F2—C34	1.3652 (16)	C22—C23	1.3892 (18)
O1—C1	1.2227 (16)	C22—H22	0.9500
O2—C12	1.3668 (14)	C23—C24	1.363 (2)
O2—C4	1.4269 (15)	C23—H23	0.9500
O3—C44	1.3623 (15)	C24—C25	1.369 (2)
O3—C5	1.4290 (17)	C25—C26	1.3860 (18)
C1—C2	1.4602 (17)	C25—H25	0.9500
C1—C11	1.5125 (15)	C26—H26	0.9500
C2—C3	1.3315 (18)	C31—C36	1.383 (2)
C2—H2	0.9500	C31—C32	1.390 (2)
C3—C41	1.4604 (17)	C32—C33	1.395 (2)
C3—H3	0.9500	C32—H32	0.9500
C4—H4A	0.9800	C33—C34	1.367 (3)
C4—H4B	0.9800	C33—H33	0.9500
C4—H4C	0.9800	C34—C35	1.364 (3)
C5—H5A	0.9800	C35—C36	1.392 (2)
C5—H5B	0.9800	C35—H35	0.9500
C5—H5C	0.9800	C36—H36	0.9500
C11—C16	1.3978 (16)	C41—C42	1.3919 (18)
C11—C12	1.4028 (15)	C41—C46	1.4093 (16)
C12—C13	1.3865 (16)	C42—C43	1.3894 (18)
C13—C14	1.3958 (16)	C42—H42	0.9500
C13—H13	0.9500	C43—C44	1.3909 (17)
C14—C15	1.3919 (16)	C43—H43	0.9500
C14—C21	1.4892 (16)	C44—C45	1.3955 (18)
C15—C16	1.3962 (16)	C45—C46	1.3724 (17)
C15—H15	0.9500	C45—H45	0.9500
C16—C31	1.4927 (16)	C46—H46	0.9500
C21—C26	1.3901 (18)		
			
C12—O2—C4	116.93 (9)	C24—C23—H23	120.7
C44—O3—C5	117.47 (11)	C22—C23—H23	120.7
O1—C1—C2	120.33 (11)	C23—C24—F1	118.70 (13)
O1—C1—C11	119.44 (11)	C23—C24—C25	122.62 (12)
C2—C1—C11	120.22 (11)	F1—C24—C25	118.68 (13)
C3—C2—C1	124.49 (11)	C24—C25—C26	118.27 (13)
C3—C2—H2	117.8	C24—C25—H25	120.9
C1—C2—H2	117.8	C26—C25—H25	120.9
C2—C3—C41	126.46 (11)	C25—C26—C21	121.37 (13)
C2—C3—H3	116.8	C25—C26—H26	119.3
C41—C3—H3	116.8	C21—C26—H26	119.3
O2—C4—H4A	109.5	C36—C31—C32	118.83 (13)
O2—C4—H4B	109.5	C36—C31—C16	120.99 (12)
H4A—C4—H4B	109.5	C32—C31—C16	119.99 (12)
O2—C4—H4C	109.5	C31—C32—C33	120.81 (15)
H4A—C4—H4C	109.5	C31—C32—H32	119.6
H4B—C4—H4C	109.5	C33—C32—H32	119.6
O3—C5—H5A	109.5	C34—C33—C32	117.92 (16)
O3—C5—H5B	109.5	C34—C33—H33	121.0
H5A—C5—H5B	109.5	C32—C33—H33	121.0
O3—C5—H5C	109.5	C35—C34—F2	118.20 (16)
H5A—C5—H5C	109.5	C35—C34—C33	123.25 (14)
H5B—C5—H5C	109.5	F2—C34—C33	118.54 (17)
C16—C11—C12	118.68 (10)	C34—C35—C36	118.18 (15)
C16—C11—C1	121.93 (10)	C34—C35—H35	120.9
C12—C11—C1	119.06 (10)	C36—C35—H35	120.9
O2—C12—C13	123.33 (10)	C31—C36—C35	120.96 (15)
O2—C12—C11	115.64 (10)	C31—C36—H36	119.5
C13—C12—C11	121.01 (10)	C35—C36—H36	119.5
C12—C13—C14	120.45 (10)	C42—C41—C46	117.60 (11)
C12—C13—H13	119.8	C42—C41—C3	119.88 (11)
C14—C13—H13	119.8	C46—C41—C3	122.52 (11)
C15—C14—C13	118.60 (10)	C43—C42—C41	122.17 (11)
C15—C14—C21	121.09 (11)	C43—C42—H42	118.9
C13—C14—C21	120.28 (10)	C41—C42—H42	118.9
C14—C15—C16	121.42 (11)	C42—C43—C44	118.86 (12)
C14—C15—H15	119.3	C42—C43—H43	120.6
C16—C15—H15	119.3	C44—C43—H43	120.6
C15—C16—C11	119.80 (10)	O3—C44—C43	124.85 (12)
C15—C16—C31	117.84 (10)	O3—C44—C45	115.07 (11)
C11—C16—C31	122.33 (10)	C43—C44—C45	120.07 (11)
C26—C21—C22	118.11 (11)	C46—C45—C44	120.31 (11)
C26—C21—C14	120.76 (11)	C46—C45—H45	119.8
C22—C21—C14	121.10 (11)	C44—C45—H45	119.8
C23—C22—C21	120.97 (13)	C45—C46—C41	120.98 (12)
C23—C22—H22	119.5	C45—C46—H46	119.5
C21—C22—H22	119.5	C41—C46—H46	119.5
C24—C23—C22	118.62 (13)		
			
O1—C1—C2—C3	−169.93 (13)	C23—C24—C25—C26	1.5 (2)
C11—C1—C2—C3	9.58 (19)	F1—C24—C25—C26	−179.01 (14)
C1—C2—C3—C41	175.54 (11)	C24—C25—C26—C21	−1.9 (2)
O1—C1—C11—C16	68.39 (16)	C22—C21—C26—C25	0.6 (2)
C2—C1—C11—C16	−111.12 (13)	C14—C21—C26—C25	178.59 (13)
O1—C1—C11—C12	−104.98 (14)	C15—C16—C31—C36	−113.39 (15)
C2—C1—C11—C12	75.50 (15)	C11—C16—C31—C36	64.74 (18)
C4—O2—C12—C13	−0.32 (18)	C15—C16—C31—C32	61.56 (18)
C4—O2—C12—C11	178.25 (12)	C11—C16—C31—C32	−120.31 (15)
C16—C11—C12—O2	−177.27 (11)	C36—C31—C32—C33	−1.6 (3)
C1—C11—C12—O2	−3.68 (16)	C16—C31—C32—C33	−176.68 (16)
C16—C11—C12—C13	1.35 (18)	C31—C32—C33—C34	0.8 (3)
C1—C11—C12—C13	174.94 (11)	C32—C33—C34—C35	1.1 (3)
O2—C12—C13—C14	178.18 (11)	C32—C33—C34—F2	179.65 (17)
C11—C12—C13—C14	−0.33 (19)	F2—C34—C35—C36	179.30 (16)
C12—C13—C14—C15	−1.26 (19)	C33—C34—C35—C36	−2.2 (3)
C12—C13—C14—C21	176.82 (12)	C32—C31—C36—C35	0.5 (2)
C13—C14—C15—C16	1.84 (19)	C16—C31—C36—C35	175.56 (14)
C21—C14—C15—C16	−176.22 (12)	C34—C35—C36—C31	1.3 (3)
C14—C15—C16—C11	−0.8 (2)	C2—C3—C41—C42	178.76 (13)
C14—C15—C16—C31	177.35 (12)	C2—C3—C41—C46	−1.5 (2)
C12—C11—C16—C15	−0.77 (18)	C46—C41—C42—C43	−0.25 (19)
C1—C11—C16—C15	−174.17 (11)	C3—C41—C42—C43	179.53 (12)
C12—C11—C16—C31	−178.86 (11)	C41—C42—C43—C44	−0.3 (2)
C1—C11—C16—C31	7.74 (18)	C5—O3—C44—C43	6.38 (19)
C15—C14—C21—C26	25.55 (19)	C5—O3—C44—C45	−172.91 (12)
C13—C14—C21—C26	−152.49 (13)	C42—C43—C44—O3	−178.15 (12)
C15—C14—C21—C22	−156.51 (13)	C42—C43—C44—C45	1.12 (19)
C13—C14—C21—C22	25.45 (19)	O3—C44—C45—C46	177.93 (11)
C26—C21—C22—C23	1.2 (2)	C43—C44—C45—C46	−1.40 (19)
C14—C21—C22—C23	−176.78 (14)	C44—C45—C46—C41	0.84 (18)
C21—C22—C23—C24	−1.6 (2)	C42—C41—C46—C45	−0.02 (18)
C22—C23—C24—F1	−179.25 (14)	C3—C41—C46—C45	−179.79 (11)
C22—C23—C24—C25	0.3 (3)		

### Hydrogen-bond geometry (Å, °)

**Table:**

*D*—H···*A*	*D*—H	H···*A*	*D*···*A*	*D*—H···*A*
C3—H3···F1^i^	0.95	2.51	3.4159 (15)	159.
C5—H5B···O1^ii^	0.98	2.54	3.3534 (18)	141.
C22—H22···O1^iii^	0.95	2.51	3.4208 (18)	161.

Symmetry codes: (i) −*x*+2, −*y*, −*z*+1; (ii) −*x*+1, *y*−1/2, −*z*+3/2; (iii) *x*, −*y*+1/2, *z*−1/2.
